# Association of Diarrhea Outcomes with Drinking Water Factors, Sanitation, Hygiene, and Malaria Practices in the Population of Béré, Chad

**DOI:** 10.3390/ijerph22101497

**Published:** 2025-09-28

**Authors:** Marie-Claire Boutrin, Marci Andersen, Zach Gately, Charis McLarty, Edirlei Santos

**Affiliations:** 1 Biological Sciences Department, School of Arts and Sciences, Oakwood University, 7000 Adventist Blvd NW, Huntsville, AL 35896, USA; 2 Global Health Department, School of Public Health, Loma Linda University, 24951 North Circle Drive, Nichol Hall, Loma Linda, CA 92350, USA

**Keywords:** drinking water, water treatment, sanitation, hygiene, hand washing, malaria, diarrhea, rural community

## Abstract

Chad, one of the poorest Sub-Saharan Central African countries, has one of the worst global diarrhea burdens. Project 21 seeks to enhance community health in the rural town of Béré, Chad but it is lacking. The study aims to determine diarrhea outcomes and associated factors, such as drinking water, malaria, sanitation and hygiene resources and practices, in Béré. A survey questionnaire was administered by trained community health workers using a random sampling method. The respondents (*n* = 484) are predominantly Nangtchéré (87%) evangelical (63%) males (88%) aged between 40–59 years old (43%) with secondary school education level (37%) or 8 years of school on average, from nuclear families (78%) with seven members on average, and of medium housing standard (56%). Drinking water treatment, transport and storage (*p* < 0.001), malaria related factors (*p* < 0.001), sanitation and hygiene practices (*p* < 0.001), children diarrhea experience, and treatment (*p* < 0.001) are predictors of diarrhea outcomes in adults. Also, factors related to drinking water transport, treatment and storage (*p* < 0.001), malaria (*p* < 0.001), health advice source (*p* < 0.001), sanitation and hygiene (*p* < 0.001), adult diarrhea experiences, and treatment (*p* < 0.001) are predictors of children diarrhea outcomes. Future interventions targeting the above factors are warranted.

## 1. Introduction

Diarrheal disease contributes greatly to the global burden of infectious diseases with 1.7 billion childhood cases occurring every year [1]. It is the second highest cause of death worldwide for children under five years old, amounting to 443,832 deaths per year [1]. It is characterized by diarrhea, a symptom defined as having three or more loose or watery bowel movements in a day, that can be caused by a range of infectious agents or other underlying conditions, such as malaria [1]. Diarrheal disease is a leading cause of malnutrition and weight loss, and most of the associated causes of death are severe dehydration and fluid loss [1]. Rehydration is a crucial part of the treatment and should include an oral rehydration solution (ORS), a mixture of clean water, sugar and salt, along with a 10–14-day zinc tablets treatment to shorten diarrhea duration and promote healthy outcomes [1]. Transmission routes include ingestion of contaminated water, foods, or drinks, and oral contact with contaminated hands or objects, or bites of Plasmodium-infected mosquitoes in the case of malaria. Global studies have shown that Africa is one of the regions most affected by fecal contamination, especially in rural locations with the lowest coverage of improved water and sanitation, resulting in high levels of diarrheal disease [2,3,4].

Diarrheal disease is a major public health issue, especially for children in Chad, one of the poorest central Sub-Saharan African countries [1,3,4]. Infectious diseases, maternal, perinatal, and nutritional deficiencies account for 66% of the populations’ total deaths, and Chad has one of the highest burdens for malaria incidence (26,152/100,000 population) and child diarrhea worldwide, the top cause of years of life lost due to premature mortality [3,4]. Only forty three percent of Chadians have access to basic drinking services, 10% experience basic sanitation, one in ten children wash their hands with soap and water, and 68% of Chadians practice open defecation nationally [5]. Poverty is prominent in rural areas, resulting in notable disparities in life expectancy, nutrition, access to healthcare, clean water and efficient water storage and treatment methods, and sanitation standards [6,7]. Only 47% of children under five with diarrhea receive ORS, and children from families of higher wealth status receive ORS treatment more often than those from a lower status [8,9]. Bad sanitation costs the Chadian government approximately USD 156 million every year due to loss of working time for individuals practicing open defecation, diarrheal disease deaths, decreased productivity of sick workforce, and increased healthcare needs of sicker communities [10,11]. This data outlines the major role of diarrheal disease in Chadian health issues due to notable deficiencies in clean water access, sanitation and hygiene standards, water storage, and treatment technologies [5].

**Figure 1:**
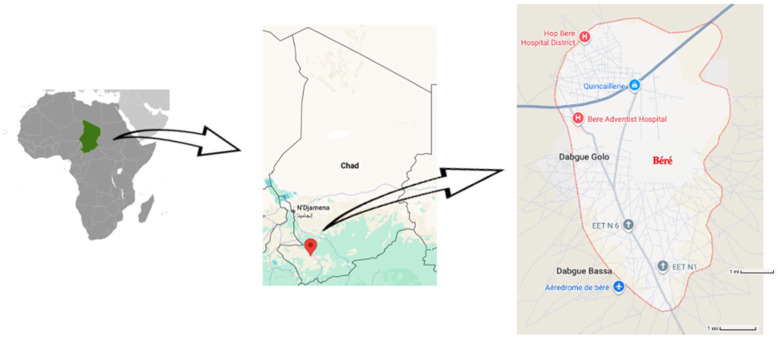
Location of Béré in Chad, Sources: https://www.cia.gov/the-world-factbook/countries/chad/locator-map/ (accessed on 1 September 2025); https://www.google.com/maps/place/Bere,+Chad/@9.3167753,16.1538742,12.62z/data=!4m6!3m5!1s0x10e092cd16fd4437:0xa7af928003dc02!8m2!3d9.316446!4d16.1610343!16s%2Fg%2F121cb1fb?entry=ttu&g_ep=EgoyMDI1MDgyNS4wIKXMDSoASAFQAw%3D%3D (accessed on 1 September 2025).

Béré, a rural city in Tandjilé, a region with the worst rates of child mortality and health issues, is found 258 miles Southeast of N’Djamena, the capital of Chad (see Figure 1). It is one the small cities in Chad and is reported to contain around 15,431 residents [12]. Its administrative infrastructure is lacking and economy relies mainly on agriculture and farming, resulting in lower socio-economic status and short education periods to prioritize work and financial security. Béré consists of twenty-one quartiers (neighborhoods) for which recent and updated population health records are not available due to decades of civil strife and resulting poverty and deficient administrative infrastructure. In addition, the precarious political and economic contexts of the country hinder health data collection and access to official healthcare institutions. Therefore, healthcare and health interventions in rural locations, such as Béré, are provided by non-governmental organizations and international entities with medical resources, such as Project 21, a community health project named after Béré’s twenty-one quartiers. The project is led in the field by public health professionals who served the community for at least a year, and aims to promote health in Béré by focusing on four main topics: community health education, midwife training with traditional birth attendants (TBAs), community health worker (CHW) training, and dental care through a dental clinic. Trained TBAs and CHWs become instrumental in prenatal and postnatal care, child deliveries, and health education events.

While Project 21 is committed to improving health in Béré, the lack of up-to-date and reliable health data from official sources has paused the planning of customized health interventions. Also, there is a notable deficit in studies on rural Chadian communities on the international scale. This study brings novel baseline data for diarrhea outcomes in Béré and an understanding of the factors contributing to diarrhea in children and adults in Béré, such as water access, storage and treatment practices, sanitation and hygiene practices, and malaria factors. To our knowledge, this is the first public health project investigating the above factors in this location in Chad. Further, this study provides possible targets for future health interventions to promote community health in Béré, and probably rural regions in other international locations, such as sub-Saharan countries, showing similar diarrheal outcomes and practices.

## 2. Materials and Methods

### 2.1. Study Setting and Design

A community-based cross-sectional study was carried out by trained CHWs and personnel in the 21 quartiers of Béré, a 27 square miles rural city found in the Béré District, in the Tandjilé area, 258 miles Southeast of N’Djamena, the capital of Chad. Due to decades of armed conflicts and subsequent poverty, Béré lacks administrative infrastructure, and its economy relies mainly on agriculture and farming. Health records are not readily available or updated. Since Project 21 focuses on improving health outcomes in Béré, quartier residents were considered the priority population for the study’s collection of data. Village chiefs were also involved in the planning and preparation of the study to promote cultural fluency and an easy facilitation of the survey.

### 2.2. Survey Respondents

Due to cultural standards and the lack of updated and reliable health data on Béré’s population, the survey was administered to household heads to collect data on their household members. Household heads who participated in the study are referred to as “respondents” in the study and include all genders, ethnicities, religious, and socio-economic backgrounds, and are at least 18 years old. Individuals who are younger than 18 years old, mentally unsound, or unable to give consent, who are not the household head nor recommended by the household head, and who lived less than 1 year in the household, were excluded from participating in the survey. One family unit is defined as one husband, wife/wives, and children, or one adult (at least 18 years old) either male or female, and children, or one single adult (at least 18 years old). A household is defined as a group of persons who share the same kitchen or hearth, or a group of persons who eat from the same cooking pot/kitchen. Households could be made of a nuclear family or a combination of co-habiting families (multi-family household). To promote good standards of sampling, only one family per multi-family household was interviewed. Respondents answered questions about all of the children in their household, since children were too young to be interviewed.

### 2.3. Sample Size Calculation

The sample size was established with the commonly used sample size PASS software version 13 for a cross-sectional study (NCSS Statistical software, Kaysville, UT, USA) using the expected malaria prevalence in children. Malaria is a leading cause of mortality in Chad, especially in children under-five at a proportion of 41% [13]. The following common parameters were used for sample size determination: 80% (0.8) power, 5% desired precision, 95% confidence interval. With these parameters, an estimated mean household size of 7, and children younger than ten years old, making 39% of the population, the minimum sample size needed to guarantee statistical power for the study was 400 households [14]. However, to promote the statistical power of the study, the number of households was increased to 517.

### 2.4. Data Collection

The simple random sampling method was used to select respondents’ houses for interview and promote sample diversity. Using a map of the city of Béré, residential houses were numbered, and house numbers were randomly chosen using an online tool (https://www.randomizer.org/, accessed on 8 June 2020). If the household head was absent at the time of the visit, permission to interview the adult in charge next to the household head was requested. If all adults were absent, the household was revisited at a time when they would be at home.

The tool used for the survey was a questionnaire based on previously validated questionnaires including Demographic and Health Survey (DHS) questionnaires that targeted demographics, malaria, water quality, treatment and storage, sanitation, deworming, and diarrhea outcomes [11,15,16,17,18,19,20,21,22]. Diarrhea was defined as the passage of three or more loose or liquid stools per day. The questionnaire was developed in English and translated and administered in French. Qualified Project 21 trainers performed the training of local CHWs following World Health Organization guidelines and protocols for teaching and carrying out survey data collection. The training of CHWs was completed as previously outlined in Boutrin et al. [23]. The questionnaires were administered by Project 21-trained CHWs who performed well and lived in or close to the quartiers of residence to promote cultural fluency and social interactions with residents. Respondents’ information was recorded using the questionnaire during household visits by trained CHWs.

### 2.5. Human Subjects’ Protection

The information collected during the survey was recorded anonymously to protect respondents. To respect the rights of individuals to participate (or not) in the survey, a verbal consent statement was read to every potential respondent to obtain their consent prior to taking the survey. Also, an IRB authorization was obtained (IRB approval #5150151) and its guidelines were followed when administering the survey and handling its data.

### 2.6. Wells Mapping

A list of pumped wells was provided by an official of Béré. Data for the well points were collected using Fulcrum (Fulcrum, San Francisco, CA, USA) for Android in the field. The polygon for the city’s boundary was created using the Esri Collector Application for Android (Esri, Redlands, CA, USA). Following data collection and synchronization, both datasets were uploaded to the Esri cloud service. From the cloud service, the point and polygon data were downloaded and opened in ArcGIS Desktop (Esri, Redlands, CA, USA) where the map was designed. The Bing satellite image was used as base map to promote high resolution.

### 2.7. Variables Included in the Study

The dependent variables in this study are diarrhea experiences in adults and children. Due to the lack of official and accurate information on diarrhea outcomes in adults and children in Béré because of decades of civil unrest, the respondents were asked about the number of adults and children in their households who experienced diarrhea in the last 12 months. Diarrhea outcomes were investigated because (1) they are known factors of weight loss and illness in adults and children, and mortality in children, and (2) previous observations from Project 21 personnel included diarrhea outcomes recurrence in Béré, (3) gaining knowledge on diarrhea outcomes and their factors in Béré will help to address the deficiency in available data and enable the development of health interventions targeting diarrhea issues.

The independent variables used in this study include known factors of diarrhea, such as socio-demographic indicators, drinking water-related indicators (e.g., treatment, storage, transport, access), sanitation and hygiene practices (e.g., stools disposal facilities and practices, handwashing), deworming practice, diarrhea cases treatment, and malaria indicators, since diarrhea is one of the symptoms of the disease and may contribute to diarrhea outcomes. The data for the variables were collected using a survey questionnaire made from validated and published questionnaires targeting these factors, as mentioned in the Section 2.4. Questions about drinking water targeted (1) its sources during dry and rainy seasons, the number of times per day respondents went to the water source for drinking water, the number of minutes taken to fetch drinking water; (2) whether water was treated; if “yes”, types of treatment; (3) how the water was stored, and (4) whether the water storage was cleaned. Sanitation and hygiene questions targeted (1) the type of toilet facility used by household adults and children; (2) whether the toilet facility was shared with other households; if “yes”, how many; (3) practices used to dispose of the stools the last time the youngest child passed stools; (4) whether handwashing was practiced by household members after going to the toilet and before eating, if “yes”, when (always or sometimes or never) and how (with water or with water and soap). Deworming questions targeted (1) whether the respondent or any household member participated in deworming programs within the last 12 months; if “yes”, how many adults and children were involved and how many times. The diarrhea case treatment question inquired how many household adults (including respondents) and children received treatment for diarrhea in the last 12 months. Malaria questions investigated (1) the number of household adults and children who experienced malaria symptoms (fever, headache, appetite loss, vomiting, convulsions, diarrhea, joint pain, sweating, and shivering) within the last 7 days, (2) the last time respondents’ mosquito nets were treated with insecticide, (3) the number of times malaria was experienced in the household within the last 12 months, (4) the number of times malaria was treated in the household within the last 12 months, (5) the source of malaria treatment, (6) practices to reduce the risk of acquiring malaria in the last 12 months, and (7) whether respondents believed that malaria is a health threat. The respondents were also asked about household characteristics (e.g., location, type (nuclear or multi-family), number of household members).

### 2.8. Data Analysis

After data collection, qualified Project 21 personnel checked the completed questionnaires for errors. Only properly filled and readable questionnaires were used for data entry and analysis. If non-response cases were found, the entire data for the relevant respondents were removed from the dataset. Following data entry, data analysis was carried out using SPSS version 27 (IBM SPSS Statistics for Windows, Armonk, NY, USA; IBM Corp.) and SAS version 19.4 (SAS Institute Inc., Cary, NC, USA).

Descriptive data analysis included frequency, means, percentages. Chi-square tests of independence were used to determine statistically significant associations between categorical variables. Statistically significant differences between means were determined using *T*-test and One-Way ANOVA test when comparing two groups and at least three groups, respectively. Linear regression was used to, respectively, identify predictors of diarrhea experiences in adults and children, two continuous variables. All independent variables were continuous variables that were added simultaneously to the model. Logistic regression analysis estimated the odds or likelihood of diarrhea outcomes for adults and children exposed to the studied factors versus those who were not, when the related variables were categorical. Collinearity between independent variables was checked through correlation matrices and VIF tests. Results are considered statistically significant at 5% levels of significance (*p* < 0.050).

## 3. Results

### 3.1. Respondents’ Demographics

A table providing a summary of the respondents’ demographics information such as household type, religion, ethnicity, house standard, age group, education level and years spent in education, household size and head gender, health advice history and source, household drinking water sources and transport, household malaria experiences, symptoms, treatment, and prevention strategies, and household diarrhea experiences, and treatment is available below (see Table 1). The respondents (*n* = 484) are household heads, predominantly Nangtchéré (87%) evangelical (63%) males (88%) aged between 40–59 years old (43%) with secondary school education level (37%) or 8 years of school on average, from nuclear families (78%) of approximately seven members on average, and of medium housing standard (56%), suggesting that over half of the respondents seem to have a medium socioeconomic status. The highest proportion of low standard houses was in Bangar (16.5%), while Nergue-Goudjba had the greatest percentage (10%) of medium standard houses and Béré Mission 1, the highest amount of high standard houses (15%). Bangar had the greatest proportion of respondents (10%) while Tchamangue had the lowest (1%).

### 3.2. Mapped Pumped Wells

**Figure 2:**
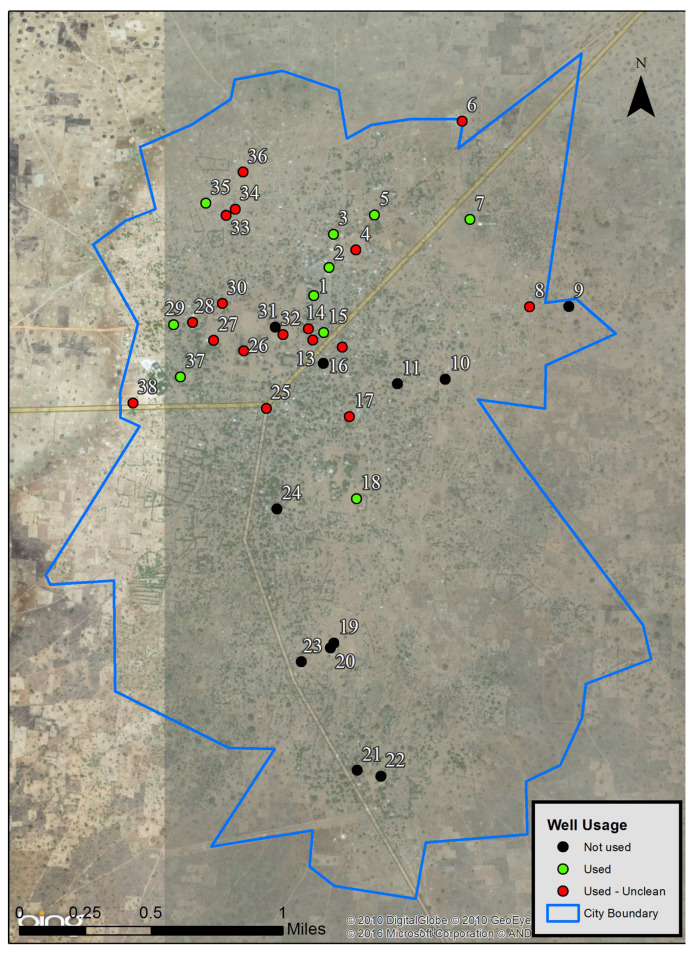
Map and description of pumped wells in Béré, Chad.

Diarrhea outcomes are known to be associated with drinking water access and quality. To gain an understanding of the public sources of drinking water available in Béré, a list of the mapped wells and their respective description is outlined in Figure 2 and Table 2. The description of the wells and their water is solely based on observations using color, taste, and physical aspect (e.g., aspect of water, presence of abnormal components, such as debris and/or oil-like layer) to form a primary report for Béré. More than a quarter (28%) of the mapped wells are not in use, some of them (10.5%) are sources of water not used for drinking, over half of them (58%) produce contaminated water (e.g., red, yellow, oily, bad taste), leaving only approximately a quarter (24%) of the wells as sources of seemingly normal water. The Béré Posté Papa Samidi well is not a true pumped well but is being referenced as such by the city’s official and residents as it is covered and produces ‘good’ water.

### 3.3. Water Sources Used by Respondents

Drinking water sources were investigated since they are possible factors in diarrhea outcomes. As shown in Table 1, most of the respondents (79%) primarily use pit wells during the dry season, whereas nearly half (49.6%) of them use rainwater and 43% use water from pit wells during the rainy season. Approximately three quarters of the respondents use uncovered pit wells during the dry and rainy seasons

### 3.4. Water Transport for Respondents

The number of times and time required to transport drinking water are known to be factors influencing the selection of water sources. The main descriptive findings on water transport are listed in Table 1.

#### 3.4.1. Number of Times per Day

A Paired *T*-test was performed to determine whether there was a difference between the number of times per day respondents’ household members went to the drinking water sources during the dry and rainy seasons. The mean number of times per day reported to go to the drinking water source is significantly lower (t(483) = 14.773; *p* < 0.001) in the rainy season (M = 5.99; SD = 4.3) than in the dry one (M = 7.8; SD = 5.3). Respondents in Nergue Bakya report the highest number of times per day (M = 12.3) used to obtain drinking water during the dry season whereas those from Béré Mouraye have the lowest (M = 3.2). In the rainy season, respondents from Nergue Bakya show the highest mean number of times (M = 9.6) used to obtain drinking water while those from Toupadjer Ngolo have the lowest mean number of trips (M = 3.6).

#### 3.4.2. Time Spent to Fetch Water

While no statistically significant difference was found between the amount of time daily spent fetching drinking water during seasons, a One-Way ANOVA test revealed that there is a statistically significantly difference in the amount of time spent fetching water between water sources for both seasons: between rainwater and pumped wells time, pumped wells and pit wells time (F(4, 479) = 4.569; *p* = 0.001) in the dry season, and between rainwater and pumped wells, pit well and pumped wells (F(4, 479) = 5.509; *p* < 0.001) in the rainy season. The longest average time spent fetching water during the dry and rainy seasons is for pumped wells (dry season M = 18.8 min; SD = 14.33; rainy season M = 21.03; SD = 18.27) and the shortest is for rainwater (dry season M = 11.10 min; SD = 8.89; rainy season M = 10.04; SD = 11.31).

#### 3.4.3. Water Carrier

The predominant water carriers reported by participants are women or wives (73.8%) in all quartiers and their average age is 25.6 years old.

### 3.5. Water Treatment Used by Respondents

A table showing descriptive findings on respondents’ drinking water storage containers and cleaning practices, drinking water treatment practices, stools disposal practices and types of facilities used, handwashing practices after toilet use and before eating a meal, and deworming practices is available below (see Table 3). Most of the respondents (86.4%) report treating their drinking water. A Chi-Square test of independence shows a significant association between water treatment and respondents quartiers (χ^2^ (20, 484) = 59.171; *p* < 0.001). The quartiers with the highest percentage of no water treatment are Béré Mission 1 (21.2%) and Nergue Annah (13.6%). The respondents’ most practiced method of treatment is bleach (64.0%) and the least practiced ones are solar disinfection (99.6%) and boiling (97.5%). Respondents from Béré Bornou, Nergue Bakya, and Singuir exclusively use bleach treatment.

### 3.6. Water Storage Conditions of Respondents

The main findings for water storage include results for storage categories and cleaning practices of water storage items, and are outlined in Table 3. The main method of storage used by respondents’ households for drinking water in Béré is terra cotta or clay-like containers (97.3%). A minimum of 92% of respondents in each quartier use this method of storage. Most of the respondents (99.6%) reported cleaning their drinking water container every week (76.9%) with clean water. Some respondents from other quartiers also stated using bleach (Nergue Bakya: 90%; Béré Bornou: 57%) and soap (Nergue Bakya: 80%; Nergue Annah: 65%) weekly to clean their water storage containers. Chi-square tests of independence show statistically significant associations between respondents’ quartiers of residence and cleaning frequency with bleach (χ^2^ (100, 484) = 212.744; *p* < 0.001), and quartiers of residence and cleaning frequency with soap (χ^2^ (80, 484) = 193.341; *p* < 0.001).

### 3.7. Sources of Health Advice

A description of the methods contributing to respondents receiving health advice is outlined in Table 1. Most (91.3%) respondents received health advice, with 77% stating the exclusive source to be CHWs, and 63% mentioning TBAs.

### 3.8. Sanitation and Hygiene Practices of Respondents

All the descriptive findings related to sanitation facilities and hygiene practices are listed in Table 3.

#### 3.8.1. Toilet Facilities Used

The toilet facilities mainly used by respondents and their household members are unimproved, with pit latrines without slab/open pits being dominant (54.9% for adults; 68.9% for children). The proportion of the types of toilet facilities used by adults and children are similar. Open defecation is reported for 4.6% of adults and 5.3% of children. One-Way ANOVA analysis shows the use of pit latrines with no slabs significantly varies with house standard (economic status indicator) for both adults and children (adults: F(2, 481) = 7.763; *p* < 0.001; children: (F(2, 481) = 14.640; *p* < 0.001)), and similar findings are obtained for open defecation (adults: F(2, 481) = 23.162; *p* < 0.001; children: (F(2, 481) = 6.989; *p* = 0.001)). Post-Hoc tests reveal that the significant differences for pit latrines with no slab use are between low and medium standards (*p* < 0.001), and medium and high standards (*p* = 0.043) for adults and children, and between low and medium standards (*p* < 0.001), low and high standards (*p* < 0.001) for children. For open defecation practice, the significant differences were identified between low and medium standards (*p* < 0.001; *p* < 0.001), low and high standards (*p* < 0.001; *p* = 0.006) for adults and children, respectively. Also, the use of pit latrines with no slab for household adults and children was significantly different between respondents’ educational levels (adults: F(4, 479) = 4.313; *p* = 0.002; children: (F(4, 479) = 7.132; *p* < 0.001)). Post-Hoc analysis shows the differences for adults are between respondents with no education and those with secondary school level education (*p* = 0.004), and between respondents with no education and those with primary (*p* < 0.001) and secondary (*p* < 0.001) school levels for children.

More than half (63.6%) of respondents’ households share their toilet facility with other households, with an average of two households, and 55% of those facilities are also accessible to the public. Chi-Square tests of independence show associations between the practice of sharing the toilet facility with other households and quartiers (χ^2^ (20, 484) = 37.915; *p* = 0.009), educational level (χ^2^ (4, 484) = 16.966; *p* = 0.002), and house standard (χ^2^ (2, 484) = 18.443; *p* < 0.001). The highest proportions of respondents sharing toilet facilities are found in Tchamangue (100%) and Nergue Bakya (90%), whereas those sharing toilet facilities, the least (25%) are in Tchouroue-Yendei. In addition, households with toilets accessible to the public are associated with respondents’ educational level (χ^2^ (4, 484) = 17.531; *p* = 0.002) and house standard (χ^2^ (2, 484) = 16.510; *p* < 0.001).

#### 3.8.2. Disposal of Children Stools

The findings on the disposal methods of children stools are shown in Table 3. The top methods used for children stools disposal are stools burial (28%), stools thrown into garbage (24%), and stools put or rinsed into toilet/latrine (20%). Chi-Square tests of independence reveal that children stools’ disposal practices are significantly associated with quartiers (χ^2^ (120, 484) = 439.458; *p* < 0.001), educational level (χ^2^ (24, 484) = 49.594; *p* = 0.002), and house standard (χ^2^ (12, 484) = 56.781; *p* < 0.001). The quartier where children predominantly use latrines is Kobtcha (53%), children’ stools are put in a toilet/latrine is Tchamangue (75%), thrown into garbage is Cotton-Tchad (69%), buried is Béré Mouraye (86%), and left in the open is Toupadjre Mbassea (100%).

#### 3.8.3. Hand Washing Practices

A description of the results about hand washing practices is included in Table 3. Most respondents (98%) practice hand washing. Hand washing with water after using the toilet is similarly practiced, whether it is “always”, “sometimes”, or not used. However, hand washing with water and soap after using the toilet is predominantly not reported (46%), and more than double the percentage of respondents state “always” washing their hands (37%) compared to those who practice it “sometimes” (16.5%). Half of the respondents mention “always” exercising hand washing with water before eating, while 45% do not practice it. In addition, most participants report hand washing with water and soap before eating (73.5%), of which more than half “always” practice it. Chi-Square test of independence shows a significant association between quartiers and all hand washing practices: hand washing with water after using the toilet and (χ^2^ (40, 484) = 178.991; *p* < 0.001), hand washing with water and soap after using the toilet (χ^2^ (40, 484) = 238.428; *p* < 0.001), hand washing with water before eating (χ^2^ (40, 484) = 149.945; *p* < 0.001), and hand washing with water and soap before eating (χ^2^ (40, 484) = 145.172; *p* < 0.001). Educational level is also significantly associated with all hand washing practices except for hand washing with water and soap after using the toilet: hand washing with water after using the toilet (χ^2^ (8, 484) = 19.792; *p* = 0.011), hand washing with water before eating (χ^2^ (8, 484) = 28.894; *p* < 0.001), hand washing with water and soap before eating (χ^2^ (8, 484) = 35.050; *p* < 0.001). Respondents house standard is only significantly associated with hand washing with water after using the toilet (χ^2^ (4, 484) = 11.264; *p* = 0.024). Hand washing with water after using the toilet and before eating is predominantly found in Singuir (70% and 89%, respectively).

### 3.9. Deworming in Respondents’ Households

The findings for deworming are listed in Table 3. Most of the respondents (82%) participated in a deworming program within the last 12 months, while 18% did not. More children were dewormed than adults (36% versus 61%), and slightly more children (M = 1.7; SD = 1.4) than adults (M = 1.2; SD = 1.3) received the treatment. One-Way ANOVA test shows that significant differences in adult and children participation in deworming programs in the last 12 months are observed between respondents’ house standards (adults: F(2, 481) = 7.008; *p* = 0.001; children: (F(2, 481) = 13.203; *p* < 0.001), with the differences being between low and medium standards (adults: *p* < 0.001, children: *p* = 0.001), and low and high standards (adults: *p* = 0.015, children: *p* < 0.001). The number of times household adults who participated in the deworming program within the last 12 months significantly vary with house standard (F(2, 481) = 5.826; *p* = 0.003) and the differences are observed between low and medium standards (*p* = 0.004), and low and high standard (*p* = 0.007). Moreover, the number of household children who participated in the deworming program differ with respondents’ educational level (F(4, 479) = 5.737; *p* < 0.001). The differences are observed between respondents with no education and those with primary (*p* = 0.014) and secondary school levels (*p* < 0.001), and secondary and baccalauréat levels (*p* = 0.030). Also, the number of times household children participated in deworming programs in the last 12 months significantly varies with respondents education level (F(4, 479) = 3.694; *p* = 0.006), with the significant differences being between respondents with baccalauréat and higher level and those with no school education (*p* = 0.004), primary school level (*p* = 0.029), and those with baccalauréat level education (*p* = 0.024).

### 3.10. Malaria Indicators

Malaria symptoms include diarrhea, suggesting a possible contribution of malaria infections to the diarrhea burden in Béré. All malaria and related indicators are presented in Table 1.

#### 3.10.1. Malaria Infections

After fever (adults: 23%); children: 31%), diarrhea is the greatest malaria symptom experienced by adults (12%) and children (28%) in Béré, followed by headache (adults: 9%; children: 19%). Respondents’ households experienced malaria on average 5.2 times in the last 12 months but received malaria treatment only 4.4 times during that time.

#### 3.10.2. Malaria Prevention

The highest percentage of respondents (23%) had mosquito nets that were treated with insecticide 6 months to 2 years ago, and 14% had theirs treated less than 6 months ago. The source of malaria treatment mostly used by respondents are the Adventist hospital (56%) and the government hospital or clinic (52%). However, 4.5% of respondents did not seek malaria treatment. The top strategy conducted to reduce the risk of acquiring malaria in the last 12 months was using insect repellent on the body. While almost all respondents (99%) believe malaria is a health threat, a third of them (33.5%) did nothing to reduce the risk of acquiring malaria in the last 12 months. Chi-Square tests of independence show that respondents’ educational level is significantly associated with using the Adventist hospital as a source of malaria treatment (χ^2^ (4, 484) = 15.500; *p* = 0.004), using body insect repellent to reduce the risk of acquiring malaria (χ^2^ (4, 484) = 11.264; *p* = 0.024) and doing nothing to lower the risk of acquiring malaria (χ^2^ (4, 484) = 14.048; *p* = 0.007). Respondents’ house standards are also significantly associated with the time when mosquito nets were last treated with insecticide (χ^2^ (6, 484) = 14.252; *p* = 0.027), using the Adventist hospital as a source of malaria treatment (χ^2^ (2, 484) = 25.105; *p* < 0.001), doing nothing to decrease the risk of acquiring malaria (χ^2^ (2, 484) = 8.159; *p* = 0.017), and using body insect repellent to reduce the risk of acquiring malaria (χ^2^ (2, 484) = 23.142; *p* < 0.001).

### 3.11. Diarrhea in Respondents’ Households

All the descriptive findings about respondents’ household diarrhea outcomes and treatment in the last 12 months are included in Table 1. Diarrhea was experienced more in children (52%) than in adults (32%), with an average of one adult (SD = 0.9) and two children (SD = 2.1) per household. The portion of household members who received diarrhea treatment (adults: 30%; children: 50.5%) was close to that who experienced diarrhea. One-Way ANOVA test indicates a significant difference in children diarrhea experience with house standard (F(2, 481) = 15.638; *p* < 0.001). Post-Hoc tests show the significant differences in diarrhea outcomes in children are between all the house standards: low and medium (*p* < 0.001), low and high standards (*p* < 0.001), medium and high standards (*p* = 0.013). Additionally, One-Way ANOVA test shows a significant difference in children diarrhea experience with respondents’ education level (F(4, 479) = 5.543; *p* < 0.001). Post- Hoc tests reveal the significant differences in children diarrhea outcomes to be between no education and primary school level (*p* = 0.014), and no education level and secondary school level (*p* < 0.001).

### 3.12. Predictors of Diarrhea Outcomes

Multiple linear regression was used to identify factors associated with or predictors of diarrhea outcomes in adults and children for the past 12 months, and several statistically significant predictors of diarrhea outcomes were identified and presented in Table 4, including drinking water transport and treatment practices, malaria symptoms and prevention practices, source of health advice, sanitation practices, diarrhea experiences and treatment. None of the independent factor variables used in the regression models were found to have high collinearity. Regression models were controlled for respondents’ gender and education years, and household head gender to neutralize the effects of gender and educational level on the associations between the dependent and independent variables.

Water treatment (*p* < 0.001), transport, and storage practices, such as number of times used to obtain drinking water per day in the dry (*p* = 0.017) and rainy seasons (*p* = 0.044), number of minutes spent fetching drinking water in the rainy season (*p* = 0.037), treating drinking water especially with bleach (*p* < 0.001) and solar disinfection (*p* = 0.037), and cleaning the water storage device (*p* = 0.045), are significant predictors for diarrhea outcomes in adults [F(13, 470) = 5.462, *p* < 0.001)]. Treating the water especially with bleach and the number of times obtaining water per day during the dry season are negatively associated with adult diarrhea outcomes. Also, malaria related factors such as fever symptoms in adults (*p* < 0.001), vomiting in adults (*p* = 0.021) and children (*p* = 0.026), headache and diarrhea symptoms in adults (*p* = 0.020), the number of times malaria was experienced (*p* = 0.006) and treated (*p* = 0.012) in the household in the last 12 months, changing the type of clothes worn (*p* = 0.008) and using house insect repellent (*p* = 0.044) to lower malaria risk are significant predictors of diarrhea outcomes in adults in Béré [F(30, 418) = 10.268, *p* < 0.001)]. In addition, sanitation and hygiene practices, such as using toilet facilities with a septic tank for adults (*p* = 0.025) and children (*p* = 0.020), pit (*p* = 0.032) and ventilated pit latrines (*p* = 0.044) for adults, pit latrine with no slab for adults (*p* = 0.003) and children (*p* = 0.044), open defecation for adults (*p* < 0.001), the number of households that share the toilet facility (*p* = 0.004), and public access to the toilet facility (*p* = 0.006), are significant predictors of diarrhea outcomes for adults [F(22, 461) = 4.829, *p* < 0.001)]. The use of improved toilet facilities with a septic tank by adults and toilet facilities accessible to the public are negatively associated with diarrhea outcomes. Further, having children with diarrhea in the same household (*p* < 0.001) and receiving treatment for diarrhea for household adults (*p* < 0.001) and children (*p* < 0.001) are significant predictors of diarrhea in adults [F(11, 472) = 341.924, *p* < 0.001)]. The number of household adults and children who received treatment for diarrhea in the last 12 months is negatively associated with adult diarrhea outcomes in the same period (see Table 4).

Regarding diarrhea outcomes in household children, factors related to water transport, treatment, and storage, such as the number of minutes spent fetching drinking water in the dry season (*p* = 0.045) and treating the drinking water, especially with bleach (*p* < 0.001), are found to be significant predictors [F(13, 470) = 7.191, *p* < 0.001)]. Except for the number of minutes spent fetching drinking water, all the other factors were negatively associated with diarrhea outcomes in children. Also, factors related to malaria, such as fever symptoms in adults (*p* = 0.021) and children (*p* < 0.001), headaches (*p* < 0.001), appetite loss (*p* < 0.001), diarrhea (*p* < 0.001), and joint pain (*p* = 0.044) symptoms in children, acquiring malaria treatment from the local market (*p* = 0.016), changing clothes (*p* = 0.022), and using insect repellent in the house (*p* = 0.001) to lower malaria risk, are significant predictors of diarrhea outcomes in children [F(30, 418) = 38.748, *p* < 0.001)]. In addition, the sources of health advice, such as CHWs only (*p* < 0.001) and TBAs only (*p* = 0.001), significantly predict children diarrhea outcomes [F(6, 477) = 11.153, *p* < 0.001)]. Moreover, factors concerning sanitation and hygiene, such as household children using pit latrines with slab (*p* < 0.001), household adults (*p* < 0.001) and children (*p* < 0.001) using pit latrines with no slab, children practicing open defecation (*p* < 0.001), the number of households sharing the toilet facility (*p* = 0.013), and toilet facility accessible to the public (*p* < 0.001), are significant predictors of children diarrhea experiences [F(22, 461) = 25.775, *p* < 0.001)]. Further, diarrhea experiences in household adults (*p* < 0.001) and the number of household adults (*p* < 0.001) and children (*p* < 0.001) who received treatment for diarrhea in the last 12 months are significant predictors of diarrhea experiences in household children [F(11, 472) = 2338.606, *p* < 0.001)]. The number of household adults who received treatment for diarrhea is negatively associated with the number of household children diarrhea experiences (see Table 4).

### 3.13. Odds Ratios for Diarrhea Outcomes in Adults and Children

Binary logistic regression was carried out to determine significant odds ratio for diarrhea outcomes in respondents’ household adults and in children, and the results are listed in Table 5, showing significance for certain drinking water sources, storage and cleaning of storage practices, mosquito net treatment practices, sanitation and handwashing practices. None of the independent factor variables used in the regression models were found to have high collinearity.

For adults, when considering water source and storage factors, the odds of experiencing diarrhea significantly decrease with acquiring drinking water from pit wells in the rainy season compared to rainwater (89% decrease in odds; OR = 0.108; 95% CI = 0.064–0.183; *p* < 0.001). The odds of experiencing diarrhea outcomes increased 17.6 times when the water storage was cleaned with water every week (compared to not being cleaned with water (*p* = 0.002)), however, cleaning of water container with soap every week (68% decrease in odds; OR = 0.320; 95% CI = 0.180–0.570; *p* < 0.001) and every month (98% decrease in odds; OR = 0.016; 95% CI = 0.001–0.324; *p* = 0.007) compared to no cleaning. Also, factors related to malaria affect the odds of household adults experiencing diarrhea. They increase when the last time the mosquito nets were treated was less than 6 months ago compared to never treating them (OR = 2.286; 95% CI = 1.228–4.257; *p* = 0.009), and decrease when the nets were treated more than 2 years ago for the last time compared to never treating them (71% decrease in odds; OR = 0.287; 95% CI = 0.110–0.750; *p* = 0.011). Further, concerning sanitation and hygiene factors, the odds of adults experiencing diarrhea are increased when children stools are thrown into the garbage compared to using a toilet or latrine (OR = 3.927; 95% CI = 1.770–8.714; *p* < 0.001), and when hand washing with water after using the toilet happens sometimes compared to not at all (OR = 4.055; 95% CI = 1.822–9.027; *p* < 0.001).

Regarding children, the odds of having diarrhea are increased by storing drinking water in terra cotta/ clay-like containers (OR = 4.772; 95% CI = 1.298–17.548; *p* = 0.019) compared to plastic ones. Obtaining drinking water from pit wells in the rainy season decrease the odds of household children experiencing diarrhea (53% decrease; OR = 0.467; 95% CI = 0.272–0.802; *p* = 0.009) compared to rainwater. Also, concerning the malaria-related factors, the odds of children having diarrhea increase when using mosquito nets that were treated with insecticide less than 6 months ago (OR = 2.700; 95% CI = 1.163–6.266; *p* = 0.021) compared to nets without treatment. Lastly, considering the sanitation and hygiene factors, the odds of children experiencing diarrhea are increased when hand washing with water after using the toilet is practiced “always” (OR = 3.709; 95% CI = 1.781–7.724; *p* < 0.001) and “sometimes” (OR = 16.060; 95% CI = 5.591–46.135; *p* < 0.001) compared to no hand washing.

### 3.14. Covariate Analysis

Additional analyses were completed to identify possible relationships between covariates. Chi-Square tests of independence reveal significant associations between house standard and respondents’ education level (χ^2^ (8, 484) = 74.779; *p* < 0.001), house standard and household head gender (χ^2^ (2, 484) = 31.421; *p* < 0.001), family type and respondents age (χ^2^ (4, 484) = 14.592; *p* = 0.004), respondents’ education level and religion (χ^2^ (24, 484) = 58.109; *p* < 0.001), respondents’ education level and ethnicity (χ^2^ (16, 484) = 66.839; *p* < 0.001), respondents’ education level and age (χ^2^ (16, 484) = 43.054; *p* < 0.001), and respondents’ education level and household head gender (χ^2^ (4, 484) = 53.713; *p* < 0.001).

## 4. Discussion

Diarrheal disease is a major issue in Chad. It represents one of the highest diarrhea burdens in children globally, and our data reflect this public health issue [3,4,24]. The aim of the study was achieved as a significant amount of information was gathered on drinking water practices (e.g., treatment, source, transport, storage), sanitation and hygiene practices (e.g., stools disposal, hand washing, toilet facility use), malaria-related indicators and practices (e.g., symptoms, treatment, prevention), diarrhea indicators (e.g., experiences and treatment), and the associations between the above practices and diarrhea outcomes in the quartiers of Béré, Chad. Diarrhea, previously reported as a disease associated with poverty, matches the economic status of the Chadian population studied here [25].

First, the map of Béré’s pumped wells provided a needed update on their location, usage, and description. The data reports a considerable shortage of working wells delivering water that looks normal. The lack of good working pumped wells would explain the predominant use of pit wells as the respondents’ main source of drinking water throughout the year and confirms previous reports of pumped wells having short longevity in use and as sources of safe water sources [26]. However, pit wells are unimproved water sources and can easily become contaminated, especially during the rainy season, where floods usually occur throughout the city and fecal bacterial contamination happens more readily, as shown in previous studies [27,28].

Second, the longer water collection time observed for pumped wells may be due to their location, functional state, crowding at the site, and the need of additional pumping at first in the morning to get rid of accumulated contaminants [26]. Also, the higher number of daily trips made to drinking water sources during the dry season seems reasonable, since hot climates would cause water evaporation, increased sweating and thirst, with no rainwater at hand to supplement water use. As in previous reports, our study showed a relationship between the time spent fetching drinking water, the number of times the water was collected, and diarrhea prevalence [29]. These findings highlight the role of prolonged exposure to contaminated water or of contaminated hands to clean water in the uptake and transmission of diarrheal pathogens, whereby the latter have additional time to grow, increase their concentration and/or accumulation in the water, worsening the infectious potential of the water [29]. In addition, since most water carriers are culturally young adult females in Béré, often wives and mothers by that age, and females are most active in the household, there is a potential microbial transmission risk from mother to children or sister to other siblings, increasing the risk of diarrhea pathogens transmission and infection, as observed in previous studies with enterobacteria and water carriers in a rural Rwandan and Nigerian communities [30,31]. Therefore, water and sanitation interventions should particularly target children, women, and young girls to prevent diarrheal disease transmission and promote maternal and child health in Béré. Further, considering that the cleaning of drinking water storage facilities with soap and treating drinking water with bleach negatively affected diarrhea prevalence in our study, cleaning treatments should be implemented throughout Béré to promote clean drinking water sources and storage, and facilitate a reduction in diarrheal disease prevalence. Improvements in the number, durability, and distribution of pumped wells would also provide better access to safe drinking water sources.

Third, drinking water treatment and storage practices require attention. In this study, bleach and solar disinfection treatments are negative predictors of diarrhea prevalence in adults and children. This is not surprising since bleach is proven to be a great antimicrobial agent, and its use would lend itself well in Béré since it is readily available there. Our findings agree with previous ones, where treatment of water with chlorine in rural Ethiopia decrease the incidence of diarrhea by 36% in under-fives and chlorine water treatment in schools in rural Kenya decreased disease incidence [32,33]. Also, our results show that cleaning water containers with water increases the odds of adult diarrhea outcomes whereas using soap decreases those odds. Therefore, the most efficient agents of decontamination, such as soap and bleach, should be used more often throughout the quartiers to promote clean water storage and consumption [34,35]. Previous reports have recognized the chlorine residual protection offered by bleach as it preserves water quality along the process of transport, storage, and consumption, and it should be promoted and implemented during water collection time [35]. In addition, water recontamination is of importance as it can occur when the container is not frequently cleaned with antimicrobial agents, or when microbes are introduced through contaminated hands or water. Contamination of clean water and recontamination of clean storage containers is a public health issue in rural households and focus should be given to improving water storage cleanliness, hand washing, and sanitation to address it [36]. Further, a five-fold increase in odds of children diarrhea outcomes was found when terra cotta or clay-like containers, the most predominant containers in Béré, are used for water storage. These containers are made of materials presenting rough and grainy surfaces where microorganisms can easily establish biofilms, and thus, it is not surprising that they would be more likely to render diarrhea outcomes. A previous study in Kenya showed that chlorination of household water along with water storage in plastic containers instead of clay pots produce better water disinfection and is more effective [37]. Therefore, future health interventions in Béré should include education on and implementation of chlorination water treatment along with replacement of clay pots with plastic ones for water storage, and cleaning of water storage with chlorine (bleach) or soap at the very minimum, to promote water disinfection and reduce the odds of diarrhea outcomes. Also, safe and durable water filtration devices that could be used for rainwater should also be considered as they would offer an alternative source of clean water, especially during the rainy season.

Fourth, the study revealed a dominant use of unimproved stools disposal facilities including pit latrines with no slab and open defecation that are shared with an average of two families. Our findings confirm previous reports on significant usage of unimproved or shared latrines; however, the percentages of unimproved toilet facilities and open defecation were lower in Béré than those reported for rural areas in Chad [4,24,38]. Open defecation practices are encouraged by a lack of facilities in the agriculture fields, the major occupation in Béré, or poverty which prevents residents from having facilities at home. The poorest communities, as found in rural Chad, were found to be 13 times more susceptible to practice open defecation than their rich counterparts [10]. Also, our results about toilet facilities and disposal of children stools practices support previous studies showing that households depending on shared facilities tend to be poorer, live in households with more young children headed by people with no formal education, and are more prone to practice open defecation or open child feces disposal, especially if toilet facilities are dirty and the user does not like sharing the facility [2,39,40,41,42]. The level of shared toilet facilities with at least two households can explain some of the diarrhea prevalence in this study, since it is identified as a predictor of diarrhea outcomes in our finding and was also shown to increase the odds of children experiencing diarrhea in previous studies [39,43]. In addition, our results reveal that the use of improved facilities, such as ventilated pit latrines and pit latrines with slabs, are predictors of diarrhea in adults and children, which may indicate that other factors, such as unhygienic conditions, can compromise sanitation in these facilities. For example, children using improved facilities can contaminate the facilities via contaminated hands or clothing while using them, and these contaminants can be transmitted to adults using the same facilities after them, resulting in diarrheal outcomes. Further, adults throwing children’ stools in the garbage to dispose of them are four times more likely to have diarrhea outcomes compared to those using toilet facilities. This may be due to fecal microbial contamination of the adults while disposing of the stools or handling the garbage can/container followed by transmission of the pathogens to self and to others, potentially resulting in infections and diarrhea outcomes. Previous studies reported similar observations, where unsafe disposal of child stools was associated with increased acute diarrhea occurrences and considerable levels of microbial contamination of water in rural Ethiopia and sub-Saharan African countries [44,45,46]. Therefore, it is crucial to develop health interventions in Béré that educate and promote sanitation and hygienic practices, including safe disposal of children’s stools and improved toilet facilities, and particularly focus on mothers living in households with five or more children (often found in Béré), since a previous study in sub-Saharan countries found them to be less likely to practice safe children’s stools disposal [45]. Also, a careful review of all the improved facilities in Béré is recommended to check the physical state of the facilities, their cleanliness, and state of function, establish a regular cleaning schedule of the facilities with antimicrobial agents, equip the facilities with proper hand washing structures, install household toilet facilities in all the quartiers, annihilate open defecation, and reduce the sharing of toilet facilities.

Fifth, deworming levels were good but could be improved, since almost a fifth of the respondents did not engage in the practice and the adults who did only did it once per year. Suboptimal participation percentages in deworming programs were also reported for other sub-Saharan countries, especially in rural locations, and should be addressed through education, and regular access to and utilization of deworming medication [47,48]. In addition, the treatment of household members who experience diarrhea could be improved, since our study indicates that not all the diarrhea cases were treated and that the number of treated diarrhea cases is a negative predictor of adult and children diarrhea outcomes (children cases treatment for adults’ outcomes and vice versa). When infected individuals remain untreated, they can facilitate a microbial reservoir that re-infect others, especially in contexts of shared unimproved toilet facilities, unsafe children’s stools disposal, open defecation, inefficient hand washing, seasonal flooding, and clean water access issues, as in Béré [49]. Therefore, education interventions on the importance of diarrhea treatment are urgently needed to decrease diarrheal pathogens reservoir and reduce transmission and infection risks in Béré.

Sixth, while respondents report washing their hands before eating and after toilet use, increased odds of diarrhea outcomes were obtained for respondents washing their hands with water after using the toilet. This handwashing practice has no antimicrobial properties and could contribute to the dissemination of fecal microbes while providing a false sense of cleanliness, facilitating microbial transmission through the spreading of contaminated water in adults and children. This is especially of concern considering the previously described unsafe stools disposal practices in the households and the resulting risk of fecal contamination and pathogen transmission between adults and children. Previous studies reported that handwashing with soap and water after using the toilet and before eating shows protective odds against diarrhea outcomes and reinforce the importance of hand washing with soap and water (rather than water only) against pathogens transmission [35,43,50]. Considering that hygiene and sanitation programs are believed to be the most cost-effective interventions globally, and hand washing with soap has the strongest impact against diarrheal outcomes, effective hygiene, hand washing, and sanitation education programs, such as Water, Sanitation, and Hygiene (WASH), should be implemented along with adequate soap supplies to improve sanitation indicators in Béré [29,35,39,44,51,52,53,54].

Lastly, our study shows the role of malaria symptoms, prevalence and prevention indicators of diarrhea as diarrhea predictors, and influencers of the odds of diarrhea outcomes in adults and children. It is unclear why some respondents with insecticide-treated mosquito nets for 6 months show increased odds of having diarrhea outcomes, other than some side effects from the insecticide on some individuals. Changing clothes, using house insect repellent, and receiving malaria treatment from the market are also predictors of diarrhea outcomes, and may indicate that the way these practices are performed may not be correct and the treatment given at the market may not be efficient, resulting in no protection against malaria, hence the diarrhea. Further, the number of times malaria was experienced in the household in the last year is a negative predictor for diarrhea outcomes, probably because of an increased immunity against malaria which lowers its infection potential and symptoms development (e.g., diarrhea). The involvement of diarrhea with malaria symptoms aligns with previous studies reporting a three-fold higher prevalence of diarrhea and vomiting in malarious children (compared with febrile controls), and 26% of children with malaria having diarrhea [55,56]. The biological mechanisms involving diarrhea in malarious patients include gut barrier damage due to intestinal microcirculation of *Plasmodium* spp., the malaria parasites, intestinal absorption impairment, sequestration of red blood cells in the gastrointestinal tract, and concomitant invasive bacterial infections with enteric Gram-negative bacteria enabled by impaired gut barrier function and increased permeability [57,58,59,60]. These facts emphasize the role that malaria plays in diarrhea outcomes in Béré and provide a rationale for including malaria testing in the diagnostic approach of patients, especially children, presenting with diarrhea in malaria-endemic locations, such as Chad.

### Limitations of the Study

The study is limited by a potential for reporting bias, due to inaccurate recall of participants, misunderstanding of the questions, participants trying to give answers they think would be pleasing to interviewers, interviewers making mistakes in data entry, and restricted statistical analysis due to the data collected. Also, although the final findings may be considered as indications for other rural areas, they may be limited to the location of Béré.

## 5. Conclusions

Taken together, the study reveals that Béré is notably affected by adult and children diarrhea outcomes that are associated with grave deficiencies in clean water sources, longer water transport, suboptimal storage conditions and treatment practices, malaria symptoms, questionable malaria treatment sources and ineffective prevention practices, toilet facilities issues, inadequate hand washing practices, and unsafe children stools disposal practices. Therefore, any future intervention should address all of the above issues by (1) educating the population, including the CHWs and TBAs on efficient and safe water treatment, transport, and storage; (2) learning adequate and efficient practices to prevent malaria infections and treat them, including locations offering effective malaria treatment; (3) understanding diarrheal disease pathogen contamination and transmission routes; (4) learning good hygiene and hand washing, such as the WASH program; and (5) providing resources and infrastructures, such as clean improved toilet facilities in each quartier to address the above issues; (6) repairing or replacing the current pumped wells, along with the addition of new rainwater filtration systems in all the quartiers, is urgently needed to complement the strategies against diarrheal diseases.

## Figures and Tables

**Table 1:** Demographic characteristics of respondents and their reported children.

Characteristics	Item	Count	Percentage (%)
**Respondents (*n* = 484); Respondents reported household adults (*n* = 1407); Respondents’ reported household children (*n* = 2084)**			
Household type	Nuclear family	379	78.3
	Multi-family	105	21.7
Religion	Seventh Day Adventist	20	4.1
	Evangelical	304	62.8
	Muslim	9	1.9
	Catholic	121	25.0
	Animist	11	2.3
	Other	7	1.4
	No religion	12	2.5
Ethnicity	Ngambay	48	9.9
	Nangtchéré	421	87.0
	Fulani	2	0.4
	Arabic	7	1.4
	Other	6	1.2
House standard	Low	85	17.6
	Medium	271	56.0
	High	128	26.4
Age group	<20 years old	3	0.6
	20–29 years old	74	15.3
	30–39 years old	161	33.3
	40–59 years old	207	42.8
	60+ years old	39	8.1
Education level	None	97	20.0
	Primary	141	29.1
	Secondary	180	37.2
	Baccalauréat	36	7.4
	Baccalauréat +	30	6.2
Education period	Number of years	mean = 8.1	(SD = 5.6)
Household head gender	Male	424	87.6
	Female	60	12.4
Household size	Number of members	mean = 6.7	(SD = 3.4)
Received health advice	*From CHW/TBA*		
	Yes	442	91.3
	No	42	8.7
	*From CHW only*		
	Yes	374	77.3
	No	110	22.7
	*From TBA only*		
	Yes	306	63.2
	No	178	36.8
Water sources	*Primary water source—Dry season*		
	Rainwater	50	10.3
	Water vendor	6	1.2
	Bottle/Bag of water	1	0.2
	Pumped well	45	9.3
	Pit well	382	78.9
	Spring	0	0
	*Primary water source—Rainy season*		
	Rainwater	240	49.6
	Water vendor	2	0.4
	Bottle/Bag of water	0	0
	Pumped well	33	6.8
	Pit well	208	43.0
	Spring	1	0.2
	*Type of pit well—Dry season*		
	Covered	124	27.2
	Uncovered	332	72.8
	*Type of pit well—Rainy season*		
	Covered	126	27.9
	Uncovered	325	72.1
	*Minutes fetching water—Dry season*	mean = 12.4	(SD = 11.2)
	*Minutes fetching water—Rainy season*	mean = 11.7	(SD = 13.2)
	*Times per day obtaining drinking water—Dry season*	mean = 7.8	(SD = 5.3)
	*Times per day obtaining drinking water—Rainy season*	mean = 5.99	(SD = 4.3)
	*Age of drinking water female carrier (years)*	mean = 25.6	(SD = 11.9)
Malaria	*Household members who experienced the following symptoms in last 7 days*		
	*Fever*		
	Adults	326	23.2 ^a^
	Children	645	31.0 ^a^
	*Headache*		
	Adults	129	9.2 ^a^
	Children	395	19.0 ^a^
	*Appetite loss*		
	Adults	112	8.0 ^a^
	Children	213	10.2 ^a^
	*Vomiting*		
	Adults	79	5.6 ^a^
	Children	265	12.7 ^a^
	*Convulsions*		
	Adults	14	1.0 ^a^
	Children	23	1.1 ^a^
	*Diarrhea*		
	Adults	172	12.2 ^a^
	Children	586	28.1 ^a^
	*Joint pain*		
	Adults	92	6.5 ^a^
	Children	8	0.4 ^a^
	*Sweating/Shivering*		
	Adults	16	1.1 ^a^
	Children	39	1.9 ^a^
	*Last time mosquito nets were treated with insecticide*		
	Less than 6 months	67	13.8
	6 months to 2 years	110	22.7
	More than 2 years	22	4.5
	Never	285	58.9
	*Number of times malaria experienced in household in last 12 months*	5.2	(SD = 5.8)
	*Number of times malaria was treated in household in last 12 months*	4.4	(SD = 4.6)
	*Source of malaria treatment:*		
	*Government hospital/Clinic*		
	Yes	251	51.9
	No	233	48.1
	*Adventist hospital*		
	Yes	271	56.0
	No	213	44.0
	*Market*		
	Yes	90	18.6
	No	394	81.4
	*No treatment*		
	Yes	22	4.5
	No	462	95.5
	*Things done to reduce malaria risk in last 12 months:*		
	*Nothing*		
	Yes	162	33.5
	No	322	66.5
	*Changed clothing*		
	Yes	74	15.3
	No	410	84.7
	*Used insect repellent on body*		
	Yes	238	49.2
	No	246	50.8
	*Used insecticide around the house*		
	Yes	33	6.8
	No	451	93.2
	*Belief malaria is a health threat*		
	Yes	478	98.8
	No	6	1.2
Diarrhea	*Household members who experienced diarrhea in last 12 months*		
	Adults	456	32.4 ^a^
		mean = 0.9	(SD = 0.9)
	Children	1083	52.0 ^a^
		mean = 2.2	(SD = 2.1)
	*Household members who received diarrhea treatment in last 12 months*		
	Adults	426	30.3 ^a^
		mean = 0.9	(SD = 0.9)
	Children	1052	50.5 ^a^
		mean = 2.2	(SD = 2.1)

^a^: percentage of demographic group; SD: standard deviation.

**Table 2:** List of mapped wells in Béré and their description.

Map ID	Name	Usage	Description
1	Singuir	Used	Works well.
2	Borno	Used	Works well.
4	Tcha-Asse Église No. 2	Used	Works well. Water is red when the well is not used.
3	Béré Mission A	Used	Works well.
5	Béré Mission B	Used	Works well.
6	Béré Mission B 2	Used	Works well. Water is red. Remote location.
7	Tcha-Asse Lycée	Used	Works well.
8	Tchirou Yendei	Used	Works well. Water is yellow and red in the morning.
9	Tchirou Yendei 2	Not used	Has not worked for 1 year.
10	Béré Yendei Église No. 7	Not used	Has not worked for nearly 1 year. Water was red.
12	Béré Borno	Used	Works well. Water is red; tastes bad in the morning.
13	Béré Borno 2	Used	Works well. Water is red; tastes bad in the morning.
14	Béré Borno ABA-Gana	Used	Works well. Water is red, oily. For washing only.
15	Béré Borno ABA-Gana 2	Used	Works well.
16	Yendei	Not used	Water is always red.
11	Yendei 2	Not used	Has not worked for 5 years. Pump not working.
17	Église No. 3	Used	Works well. Water is red in the morning.
18	École des Filles	Used	Works well. At a girls’ school.
19	Nergue-Goujiba École	Not used	Pump is not working.
20	Nergue-Goujiba École 2	Not used	Pump is not working.
21	Nergue-Goujiba	Not used	Has not worked for 7–8 years.
22	Nergue-Goujiba Église	Not used	Has not worked for 7 years.
23	Tchamangue	Not used	Has not worked for 2 years. Water was good.
24	Église Mission Bangar No. 4	Not used	Has not worked for 2 years. Water was red.
25	Esthar Singuir	Used	Works well. Water is red in the morning.
26	Singuir Prefecture	Used	Works well. Water is red in the evening.
27	District Santé	Used	Works well. Water is always yellow. For cooking only.
28	Singuir Mere	Used	Works well. Water is red in the morning.
29	Dàma Béré Poste	Used	Works well. Water is red in the morning.
30	Mission Béré Bolo	Used	Works well. Water is always red, oily taste. Not for drinking.
31	Sous-préfecture de Béré	Not used	Has not worked for 2 years. Water was red.
32	Sous-préfecture de Singuir	Used	Works well. Water is always red.
33	Béré Bolo Kadamou	Used	Works well. Water is yellow in the morning; looks normal after.
34	Béré Bolo Kadamou 2	Used	Works well. Water is red in the morning.
35	Béré Bolo Kadamou 3	Used	Works well. Pumping takes a long time.
36	Béré Mission A Guedetague	Used	Works well. Water is red in the morning but looks normal after
37	Béré Posté Papa Samidi	Used	Works well. Water looks normal. Not true pumped well.
38	Béré Posté École	Used	Pumping takes time. Water does not look normal. Not for drinking.

**Table 3:** Water storage and treatment, sanitation, and hygiene practices in Béré, Chad.

Practices	Item	Count	Percentage (%)
**Respondents (*n* = 484); Respondents reported household adults (*n* = 1407); Respondents’ reported household children (*n* = 2084)**			
Water storage	*Container type*		
	Plastic container	12	2.5
	Terra cotta/Clay-like container	471	97.3
	Other	1	0.2
	*Container cleaned*		
	Yes	482	99.6
	No	2	0.4
	*Cleaning frequency with water*		
	No	104	21.5
	Every week	372	76.9
	Every month	8	1.7
Water treatment	*Water treated*		
	Yes	418	86.4
	No	66	13.6
	*Treatment—Boiled*		
	Yes	12	2.5
	No	472	97.5
	*Treatment—Bleach*		
	Yes	310	64.0
	No	174	36.0
	*Treatment—Solar disinfection*		
	Yes	2	0.4
	No	482	99.6
Sanitation	Type of toilet facility used by household		
	Improved facilities		
	*Flush to septic tank*		
	Adults	11	0.8 ^a^
	Children	8	0.4 ^a^
	*Flush to pit latrine*		
	Adults	34	2.4 ^a^
	Children	44	2.1 ^a^
	*Flush elsewhere*		
	Adults	3	0.2 ^a^
	Children	14	0.7 ^a^
	*Flush to unknown place*		
	Adults	1	0.1 ^a^
	Children	0	0.0 ^a^
	*Ventilated pit latrine*		
	Adults	14	1.0 ^a^
	Children	27	1.3 ^a^
	*Pit latrine with slab*		
	Adults	146	10.4 ^a^
	Children	256	12.3 ^a^
	*Composting toilet*		
	Adult	2	0.1 ^a^
	Children	4	0.2 ^a^
	Unimproved facilities		
	*Bush/Field/No facilities*		
	Adults	65	4.6 ^a^
	Children	110	5.3 ^a^
	*Pit latrine without slab*		
	Adults	772	54.9 ^a^
	Children	1435	68.9 ^a^
	*Toilet facility shared with other households*		
	Yes	308	63.6
	No	176	36.4
	*Number of households sharing toilet facility*	mean = 2.0	(SD = 2.5)
	*Public has access to use toilet*		
	Yes	267	55.2
	No	217	44.8
	*Disposal method of youngest child stools*		
	Child used toilet/latrine	63	13.0
	Put/Rinsed stools into toilet/latrine	97	20.0
	Put/Rinsed stools in drain/ditch	1	0.2
	Stools thrown into garbage	115	23.8
	Stools buried	136	28.1
	Stools left in open	44	9.1
	Other	28	5.8
Hand washing	*Handwashing practiced in household*		
	Yes	444	98.4
	No	7	1.6
	*After using toilet—With water*		
	Always	160	33.1
	Sometimes	171	35.3
	No	153	31.6
	*After using toilet—With water and soap*		
	Always	181	37.4
	Sometimes	80	16.5
	No	223	46.1
	*Before eating—With water*		
	Always	245	50.6
	Sometimes	23	4.8
	No	216	44.6
	*Before eating—With water and soap*		
	Always	186	38.4
	Sometimes	170	35.1
	No	128	26.4
Deworming	*Household members participated in deworming program in last 12 months*		
	Yes	397	82.0
	No	87	18.0
	*Household members who participated in deworming programs in last 12 months*		
	Adults	504	35.8 ^a^
	Children	1269	60.9 ^a^
	*Number of times members participated in deworming*		
	Adults	mean = 1.2	(SD = 1.3)
	Children	mean = 1.7	(SD = 1.4)

^a^: percentage of demographic group. SD: standard deviation.

**Table 4:** Statistically significant predictors of diarrhea experiences in adults and children.

Factors	Unstandardized B	95% CI	*p*
Adults			
Times per day obtaining drinking water—Dry season	−0.037	−0.068–(−0.007)	0.017
Times per day obtaining drinking water—Rainy season	0.038	0.001–0.075	0.044
Minutes fetching drinking water—Rainy season	0.011	0.001–0.021	0.037
Drinking water treated	−0.630	−0.922–(−0.337)	<0.001
Drinking water treatment—Bleach	−0.458	−0.672–(−0.244)	<0.001
Drinking water treatment—Solar disinfection	−1.318	−2.555–(−0.082)	0.037
Drinking water storage cleaned	1.280	0.029–2.531	0.045
Fever (malaria)—Adults	0.392	0.291–0.492	<0.001
Headache (malaria)—Adults	0.177	0.028–0.325	0.020
Vomiting (malaria)—Adults	0.241	0.036–0.447	0.021
Vomiting (malaria)—Children	−0.107	−0.200–(−0.013)	0.026
Diarrhea (malaria)—Adults	0.203	0.059–0.347	0.006
Number of times malaria experienced in household in last 12 months	−0.033	−0.059–(−0.007)	0.006
Number of times malaria treated in household in last 12 months	0.040	0.006–0.073	0.012
Action to lower malaria risk—Changed clothes	0.315	0.085–0.546	0.008
Action to lower malaria risk—Used house insect repellent	0.353	0.010–0.696	0.044
Used toilet facility with a septic tank—Adults	−2.260	−4.228–(−0.292)	0.025
Used toilet facility with a septic tank—Children	3.250	0.508–5.993	0.020
Used pit latrine—Adults	0.283	0.025–0.542	0.032
Used ventilated pit latrine—Adults	0.771	0.022–1.520	0.044
Used pit latrine with no slab—Adults	0.134	0.046–0.221	0.003
Used pit latrine with no slab—Children	0.036	0.005–0.067	0.024
Used bush or field/No toilet facility—Adults	0.477	0.205–0.750	<0.001
Number of households that share the toilet facility	0.057	0.018–0.097	0.004
Toilet facility can be used by the public	−0.394	−0.677–(−0.112)	0.006
Experienced diarrhea in last 12 months—Children	0.285	0.190–0.380	<0.001
Received diarrhea treatment in last 12 months—Adults	0.953	0.915–0.990	<0.001
Received diarrhea treatment in last 12 months—Children	−0.285	−0.382–(−0.188)	<0.001
Children			
Minutes fetching drinking water—Dry season	0.028	0.001–0.054	0.045
Drinking water treated	−2.054	−2.709–(−1.400)	<0.001
Drinking water treatment—Bleach	−1.427	−1.906–(−0.948)	<0.001
Fever (malaria)—Adults	0.186	0.028–0.344	0.021
Fever (malaria)—Children	0.618	0.531–0.705	<0.001
Headache (malaria)—Children	0.237	0.126–0.348	<0.001
Appetite loss (malaria)—Children	0.426	0.267–0.584	<0.001
Diarrhea (malaria)—Children	0.363	0.266–0.460	<0.001
Joint pain (malaria)—Children	0.881	0.024–1.738	0.044
Source of malaria treatment—Market	0.428	0.082–0.775	0.016
Action to lower malaria risk—Changed clothes	0.422	0.060–0.783	0.022
Action to lower malaria risk—Used house insect repellent	0.881	0.344–1.418	0.001
CHW gave health advice	1.017	0.471–1.564	<0.001
TBA gave health advice	0.703	0.280–1.126	0.001
Used pit latrine—Children	0.344	0.025–0.664	0.035
Used pit latrine with slab—Children	0.320	0.185–0.455	<0.001
Used pit latrine with no slab—Adults	−0.334	−0.483–(−0.186)	<0.001
Used pit latrine with no slab—Children	0.491	0.438–0.544	<0.001
Used bush or field/No toilet facility—Children	0.403	0.190–0.616	<0.001
Number of households that share the toilet facility	0.085	0.018–0.152	0.013
Toilet facility can be used by the public	−1.008	−1.488–(−0.529)	<0.001
Experienced diarrhea—Adults	0.240	0.160–0.321	<0.001
Received diarrhea treatment in last 12 months—Adults	−0.252	−0.335–(−0.168)	<0.001
Received diarrhea treatment in last 12 months—Children	1.000	0.981–1.018	<0.001

Model controlled for respondents’ gender and education years, and household head gender.

**Table 5:** Statistically significant odds ratios for diarrhea outcomes in adults and children.

Factors	Odd Ratios	95% CI	*p*
Adults			
Drinking water from pit well—Rainy season	0.108	0.064–0.183	<0.001
Drinking water from rainwater—Rainy season	1 (reference)	-	
Cleaning of drinking water container with water—Every week	17.561	2.938–104.955	0.002
No cleaning of drinking water container with water	1 (reference)	-	
Cleaning of drinking water container with soap—Every week	0.320	0.180–0.570	<0.001
Cleaning of drinking water container with soap—Every month	0.016	0.001–0.324	0.007
No cleaning of drinking water container with soap	1 (reference)	-	
Last time mosquito nets were treated with insecticide—Less than 6 months ago	2.286	1.228–4.257	0.009
Last time mosquito nets were treated with insecticide—More than 2 years ago	0.287	0.110–0.750	0.011
Last time mosquito nets were treated with insecticide—Never	1 (reference)	-	
Children stools disposal—Thrown in garbage	3.927	1.770–8.714	<0.001
Children stools disposal—Toilet/Latrine	1 (reference)	-	
Hand washing with water after using toilet—Sometimes	4.055	1.822–9.027	<0.001
Hand washing with water after using toilet—None	1 (reference)	-	
Children			
Storage of drinking water—Terra cotta/Clay-like container	4.772	1.298–17.548	0.019
Storage of drinking water—Plastic container	1 (reference)	-	-
Drinking water from pit well—Rainy season	0.467	0.272–0.802	0.009
Drinking water from rainwater—Rainy season	1 (reference)	-	-
Last time mosquito nets were treated with insecticide—Less than 6 months ago	2.700	1.163–6.266	0.021
Last time mosquito nets were treated with insecticide—Never	1 (reference)	-	
Hand washing with water after using toilet—Always	3.709	1.781–7.724	<0.001
Hand washing with water after using toilet—Sometimes	16.060	5.591–46.135	<0.001
Hand washing with water after using toilet—None	1 (reference)	-	

## Data Availability

The dataset generated by the survey research during and/or analyzed during the current study is available in the Zenodo repository: https://doi.org/10.5281/zenodo.16561889.

## Abbreviations

The following abbreviations are used in this manuscript:

TBA	Traditional birth attendant
CHW	Community health worker
CDC	Centers for Disease Control and Prevention
WHO	World Health Organization
